# 

**Published:** 1911-09

**Authors:** 


### CONTENTS.

ORIGINAL COMMUNICATIONS—

Etiology, Pathology and Treatment of Pellagra. By Dr. George C. Mizell, M. D. Ph.D., Atlanta, Ga. .......... 285

Treatment of Typhoid with Remedies Other Than Drugs. By M. McH. Hull, M. D., Atlanta, Ga. .......... 306

The Gall Bladder. By J. L. Campbell, M. D. .......... 314

The Classification of Pellagra. By Hansell Crenshaw, M. D., Atlanta, Ga. .......... 320

Epilepsy. By James M. Brawner, M. D., Atlanta, Ga. .......... 323

Luetic Meningitis of the Anterio Cranial Fossa with Uncinate Gyrus Syndrome Simulating Neoplasm. By Tom A. Williams, M. B. C. M., Washington, D. C. .......... 328

EDITORIALS .......... 332

NEWS AND NOTES .......... 335

BOOK REVIEWS .......... 343

